# Exposure to Ti4Al4V Titanium Alloy Leads to Redox Abnormalities, Oxidative Stress, and Oxidative Damage in Patients Treated for Mandible Fractures

**DOI:** 10.1155/2018/3714725

**Published:** 2018-06-14

**Authors:** Jan Borys, Mateusz Maciejczyk, Bożena Antonowicz, Adam Krętowski, Danuta Waszkiel, Piotr Bortnik, Katarzyna Czarniecka-Bargłowska, Magdalena Kocisz, Julita Szulimowska, Marek Czajkowski, Napoleon Waszkiewicz, Anna Zalewska

**Affiliations:** ^1^Department of Maxillofacial and Plastic Surgery, Medical University of Bialystok, Sklodowskiej M.C. 24, 15-274 Bialystok, Poland; ^2^Department of Physiology, Medical University of Bialystok, Mickiewicza 2c Str., 15-233 Bialystok, Poland; ^3^Department of Oral Surgery, Medical University of Bialystok, Sklodowskiej M.C. 24a Str., 15-276 Bialystok, Poland; ^4^Clinical Research Centre, Medical University of Bialystok, Sklodowskiej M.C. 24a Str., 15-276 Bialystok, Poland; ^5^Department of Conservative Dentistry, Medical University of Bialystok, Sklodowskiej M.C. 24a Str., 15-276 Bialystok, Poland; ^6^Department of Pedodontics, Medical University of Bialystok, Sklodowskiej M.C. 24a Str., 15-276 Bialystok, Poland; ^7^IPL Marek Czajkowski, Aleja Solidarności 6/77 Str., 15-751 Białystok, Poland; ^8^Department of Psychiatry, Medical University of Bialystok, Brodowicza 1, 16-070 Choroszcz, Poland

## Abstract

Due to the high biotolerance, favourable mechanical properties, and osseointegration ability, titanium is the basic biomaterial used in maxillofacial surgery. The passive layer of titanium dioxide on the surface of the implant effectively provides anticorrosive properties, but it can be damaged, resulting in the release of titanium ions to the surrounding tissues. The aim of our work was to evaluate the influence of Ti6Al4V titanium alloy on redox balance and oxidative damage in the periosteum surrounding the titanium miniplates and screws as well as in plasma and erythrocytes of patients with mandibular fractures. The study included 31 previously implanted patients (aged 21–29) treated for mandibular fractures and 31 healthy controls. We have demonstrated increased activity/concentration of antioxidants both in the mandibular periosteum and plasma/erythrocytes of patients with titanium mandibular fixations. However, increased concentrations of the products of oxidative protein and lipid modifications were only observed in the periosteum of the study group patients. The correlation between the products of oxidative modification of the mandible and antioxidants in plasma/erythrocytes suggests a relationship between the increase of oxidative damage at the implantation site and central redox disorders in patients with titanium miniplates and screws.

## 1. Introduction

Jaw bone fractures are a frequent and significant problem in clinical practice. The most common types of injuries are mandibular fractures that lead to morphological and functional disorders of the stomatognathic system as well as aesthetic defects of the facial skeleton. The treatment of mandibular fractures is aimed at reconstructing the anatomical shape of the bones and occlusion from before the injury and restoring normal functions of the chewing organ and facial aesthetics [1]. The use of internal bone fixation systems with miniplates and screws allows not only to avoid long-term intermaxillary immobilization and dental splint insertion, which are troublesome for the patient, but also to cure fractures in case of contraindications for inoperative orthopaedic treatment [1]. Miniplates and screws made of titanium, due to the high biocompatibility of this metal and its alloys used in the production of such fixations, are commonly used in traumatology, maxillofacial oncology, and orthognathic surgery. Despite the undoubted advantages of titanium bone fixations, their negative impact on the human body, both at the implant site and at the systemic level, is still debated [1, 2]. During the removal of titanium bone fixations, the presence of discoloured gray tissues surrounding the miniplates and screws was often observed [3, 4]. Some patients have also suffered from inflammatory symptoms around the anastomosis sites reported postoperatively both after several months and even years after osteosynthesis of the fractured mandible. Therefore, it is important to explore the impact of titanium miniplates and screws on the surrounding tissues [2]. The results of recent studies indicate that the cause of chronic inflammation around titanium bone fixations may be the increased production of oxygen-free radicals and reactive nitrogen species (RNS) [3, 5–7]. It is believed that overproduction of reactive oxygen species (ROS) and RNS can lead to the damage of cellular components by oxidation, thereby impairing normal cell functioning as a result of increased synthesis of proinflammatory mediators and affecting growth, differentiation, and apoptosis processes in the cells [8].

Although oxidative stress is one of the mechanisms of titanium toxicity [9], the kind (and extent) of oxidative damage it can cause is still unknown. What is more, the literature on the subject so far has not offered any study evaluating the effect of titanium bone fixation systems in patients with mandibular fractures on the redox balance both at the implant site and at the systemic level. Therefore, the aim of our work was to evaluate enzymatic and nonenzymatic antioxidant systems and oxidative damage of proteins, lipids, and DNA in the periosteum surrounding the bone fixations as well as in plasma and erythrocytes of patients treated for mandible fractures, compared to the control group.

## 2. Materials and Methods

### 2.1. Patients

The protocol of the study was approved by the Bioethics Committee of the Medical University of Bialystok, Poland (permission number R-I-002/3/2-16).

62 patients operated at the Department of Maxillofacial and Plastic Surgery, Medical University in Bialystok, Poland, were enrolled in the study. The study group included 31 previously implanted patients (11 women and 20 men aged 21–29, mean age: 24 years and 7 months) treated for mandibular fractures. The causes of the fractures in patients were beating (61.2%), sports (19.4%), traffic accident (12.9%), and fall from the stairs (6.5%). All patients were treated for bilateral fracture of the mandibular body. The fractured bone fragments in the mandible were fixed with two 4- or 6-hole miniplates and 4–6 screws (on the right and left side): in total 4 miniplates and 16–24 screws (MEDGAL Sp. z o.o., Księżyno, Poland).

Two months after implantation (every 2 weeks), and then, every month until the fixations were removed, the patients went to the hospital for follow-up examinations performed by one qualified surgeon (J.B.). In all patients from the study group, there were no signs of acute and chronic inflammation such as edema, inflammatory infiltration, purulent fistula, and redness of the mucous membrane or skin around the titanium fixations. In the physical examination, no enlargement of regional lymph nodes was also observed, which did not require specialized diagnostics in these group of patients. In the study group, there were also no clinical allergy signs like edema, changes on the skin, and mucous membranes.

The control group, selected by sex and age to match the study group, consisted of 31 generally healthy patients (11 women and 20 men aged 21–29, mean age: 24 years and 2 months) operated due to dentofacial deformities, whose periosteum and blood samples were taken immediately prior to the insertion of titanium implants.

#### 2.1.1. Inclusion Criteria: Study Group

- Maxillary bone fixations after treatment for fractures of the mandibular body.

#### 2.1.2. Inclusion Criteria: Control Group

- Generally healthy patients with dentofacial deformities before surgical treatment.

#### 2.1.3. Inclusion Criteria: Study and Control Group

- Written informed consent for participation in the study,
- Age 21–29,
- 18.5 ≤ BMI ≥ 24.5,
- No former treatment for bone fracture or jaw osteotomies performed with titanium fixations,
- A noninflammation-induced healing process from insertion of fixations until their removal,
- No craniocerebral traumas (hematomas),
- Normal results of complete blood count (WBC 4.5–8.5 ×10^3^/*μ*L; RBC 3.6–5.4 ×10^12^/L; HGB 12.0–16.0 g/dL; PLT 100–450 ×10^9^/L; hematocrit 36–54%) and biochemical blood tests (CRP 0.1–5 mg/L; Na^+^ 135.0–145.0 mmol/L; K^+^ 3.8–5.0 mmol/L; international normalized ratio (INR), 0.9–1.3; activated partial thromboplastin time (APTT), 37.0–46.0 sec; prothrombin time (PT), 12.0–18.0 sec),
- No systemic (insulin resistance, diabetes, hypertension, and coronary heart disease) and autoimmune disorders (psoriasis, multiple sclerosis, and rheumatoid arthritis), hyperlipidemia, liver, kidney, thyroid, lung, gastrointestinal and infectious (HCV and HIV infection) diseases, or gingivitis, periodontitis, and active odontogenic infection foci,
- Nonsmokers,
- Nonalcohol drinkers,
- No intake of antibiotics, nonsteroidal anti-inflammatory drugs (NSAIDs), glucocorticosteroids, dietary supplements, and vitamins.

#### 2.1.4. Exclusion Criteria: Study and Control Group

- Another type of mandibular fracture (singular fracture, fracture of mandibular ramus or condyle),
- Wounds of soft tissues, injury of the skull, brain, chest, abdomen, and extremities,
- Inflammatory complications and jaw synostosis disorders after implantation,
- Operations due to bone fractures in the past and for other reasons during the last year.

The clinical parameters of patients and control group are presented in Table 1.

### 2.2. Surgical Procedure

All patients were operated by the same qualified maxillofacial surgeon (J.B.). Two months before the surgery, patients in both groups were on a diet (2000 kcal, including 55% carbohydrates, 30% fat, and 15% protein) determined by a dietician. Miniplates and screws were removed under local anaesthesia of 2% lignocaine and epinephrine (Polfa, Warsaw, Poland) from 3 to 8 months (approx. 5 months) after the surgery. The research material was a gray-pigmented periosteum adhering to the titanium miniplates excised as a standard procedure during the removal of mandibular bone fixations. In the control group, healthy periosteum was taken separately from the mandible during bimaxillary osteotomy before implantation of the miniplates and screws. Gray-pigmented periosteum was aseptic (data not shown). The tissues were immediately frozen upon collection in liquid nitrogen and stored at −80°C until assayed.

### 2.3. Blood (Plasma and Erythrocytes)

Before the surgery and after an overnight fast, 9 mL of venous blood samples were collected in plastic EDTA tubes (S-Monovette® K3 EDTA blood collection system, Sarstedt). To separate plasma samples and erythrocytes, the samples were centrifuged at 1500 ×g (4°C, 10 min; MPW 351, MPW Med. Instruments, Warsaw, Poland). Erythrocytes were washed three times in cold solution of 0.9% NaCl (w : v) and haemolysed by the addition of cold 50 mM phosphate buffer (pH 7.4) 1 : 9 (v : v) [10]. In order to prevent sample oxidation, 0.5 M butylated hydroxytoluene (Sigma-Aldrich, Germany; 100 *μ*L/10 mL sample) was added [11]. All samples were stored at –80°C until use.

### 2.4. Tissue Homogenates

Before the biochemical determinations, fragments of periosteum were rinsed in ice-cold PBS (20 mM, pH 7.0), weighed and milled into small pieces that were homogenized on ice (Omni TH, Omni International, Kennesaw, GA, USA) in ice-cold PBS (1 : 15, w : v). 0.5 M butylated hydroxytoluene (10 *μ*L/1 mL PBS) and the protease inhibitor (Complete Mini Roche, France; 1 tablet/10 mL PBS) were added to all the samples [12]. The tissue suspensions were sonicated on ice with an ultrasonic cell disrupter (UP 400S, Hielscher, Teltow, Germany; 1800 J/sample, 20 s *x* 3) and centrifuged (3500 ×g, 4°C, 20 min), and the resulting supernatants were analyzed on the same day.

### 2.5. Antioxidant Assays

Antioxidant enzymes (glutathione peroxidase (GPx), catalase (CAT), and superoxide dismutase-1 (SOD)) and total protein were analyzed in erythrocytes and tissue homogenates, while nonenzymatic antioxidants (reduced glutathione (GSH) and uric acid (UA)) and total protein were estimated both in plasma and tissue homogenates. The absorbance in all assays was measured with Infinite M200 PRO Multimode Microplate Reader, Tecan. The assays were performed in duplicates, except for the CAT (triplicate samples). Reagents for all the assays were obtained from Sigma-Aldrich, Germany (unless noted otherwise).

The activity of GPx (EC 1.11.1.9) was assessed colorimetrically by measuring the decrease in absorbance at 340 nm wavelength as a result of NADPH (chemically reduced form of nicotinamide adenine dinucleotide phosphate) oxidation. One unit of GPx activity was defined as the amount of the enzyme needed to catalyze the oxidation of 1 mmol NADPH per minute [13]. The results were expressed in units per mg of total protein.

The activity of CAT (EC 1.11.1.6) was determined colorimetrically by measuring the rate of hydrogen peroxide (H_2_O_2_) degradation at 240 nm [14]. One unit of CAT activity was defined as the amount of the enzyme needed to catalyze the decomposition of 1 mmol H_2_O_2_ per minute. CAT determination was performed in triplicate samples and the results were expressed in nmol H_2_O_2_ per mg of total protein.

The activity of SOD (EC 1.15.1.1) was analyzed colorimetrically by measuring the inhibition of adrenaline oxidation at 480 nm. One unit of SOD activity was defined as the amount of the enzyme needed to inhibit adrenaline oxidation by 50% [15]. The results were expressed as mU per mg of total protein.

The concentration of GSH was estimated colorimetrically by reaction with DTNB [5,5′-dithiobis-(2-nitrobenzoic acid)] to obtain a product with absorption maximum at 412 nm [16]. The results were expressed as *μ*g per mg of total protein.

The concentration of UA was measured colorimetrically at 490 nm by reaction with 2,4,6-tripyridyl-s-triazine using the commercial kit QuantiChromTM Uric Acid Assay Kit DIUA-250 (BioAssay Systems, Hayward, CA, USA). The results were expressed as *μ*g per mg of total protein.

The concentration of total protein was determined colorimetrically using the commercial kit Thermo Scientific PIERCE BCA Protein Assay (Rockford, IL, USA) with bovine serum album as a standard.

### 2.6. Total Antioxidant/Oxidant Status

Total antioxidant/oxidant status (total antioxidant capacity (TAC), ferric reducing ability of plasma (FRAP), and total oxidant status (TOS)) and total protein content were analyzed both in the plasma and tissue homogenates. The absorbance in all assays was measured with Infinite M200 PRO Multimode Microplate Reader, Tecan. All assays were performed in duplicate samples, excluding the TAC and TOS determination (triplicate samples).

The concentration of TAC was estimated at 660 nm by ABTS + [2,2′-azino-bis-(3-ethylbenzo-thiazoline-6-sulfonate)]-based colorimetric micromethod [17]. TAC determination was performed in triplicate samples, and the results were expressed as *μ*mol Trolox equivalent per mg of total protein.

The concentration of TOS was estimated bichromatically (560/800 nm) based on the ferrous ion oxidation to ferric ion in the presence of oxidants in the sample [18]. TOS determination was performed in triplicate samples, and the results were expressed in *μ*mol H_2_O_2_ equivalent per mg of total protein.

Oxidative stress index (OSI) was expressed in % according to the formula: OSI = TOS/TAC × 100% [19].

The concentration of FRAP was analyzed colorimetrically at 593 nm, measuring the ferric reducing ability of the sample by reaction with 2,4,6-tripyridyl-s-triazine [20]. The results were expressed in *μ*mol per mg of total protein.

### 2.7. Oxidative Damage Determination

Oxidative damage products (advanced glycation end products (AGEs), advanced oxidation protein products (AOPP), 4-hydroxynonenal (4-HNE) protein adducts, and 8-hydroxydeoxyguanosine (8-OHdG)) and total protein content were analyzed both in plasma and tissue homogenates. The absorbance/fluorescence in all assays was measured with Infinite M200 PRO Multimode Microplate Reader, Tecan. All the assays were performed in duplicate samples.

The levels of AOPP were assessed colorimetrically at 340 nm by measuring the total iodide ion oxidizing capacity of the sample [21]. The results were expressed in *μ*mol per mg of total protein.

The content of AGE was determined fluorimetrically at 350/440 nm in 1 : 5 diluted plasma samples and tissue homogenates [21]. The results were expressed in fluorescence per mg of total protein.

The concentration of 4-HNE protein adducts as well as 8-OHdG was measured with the commercial ELISA kit according to the manufacturer's instructions (OxiSelect™ HNE Adduct Competitive ELISA, Cell Biolabs Inc., San Diego, CA, USA; USCN Life Science, Wuhan, China, resp.). The results were expressed in *μ*g/pg per mg of total protein.

### 2.8. Statistical Analysis

Statistical analysis was performed using the Statistica 10.0 system (StatSoft, Cracow, Poland) and GraphPad Prism (GraphPad Software, La Jolla, USA). The D'Agostino-Pearson test and Shapiro-Wilk test were used to examine normal distribution. Since most of the data were normally distributed, the results were presented as a mean, standard deviations, geometric mean, and 95th confidence intervals of mean. In the case of normal distribution of the results, a Student's *t*-test was used. In the lack of normal distribution of the results, the Mann–Whitney *U* test was used. The associations between the measured parameters were analyzed by Pearson's correlation. The results were assumed to be statistically significant when *p* < 0.05.

## 3. Results

### 3.1. Antioxidant Defence

CAT activity was significantly lower in mandibular periosteum and considerably higher in erythrocytes of the study group compared to the control group. SOD activity was significantly higher in patients with a mandibular fracture compared to the controls, while no statistically relevant changes were observed in erythrocytes. The concentration of UA was significantly higher both in mandibular periosteum and plasma of the study group patients than in the controls. The activity of GPx in mandibular periosteum and erythrocytes and GSH concentration in mandibular periosteum and plasma showed similar values in patients in the study and control groups (Tables 2 and 3).

### 3.2. Total Antioxidant/Oxidant Status

There was a statistically significant increase in TAC level in the mandibular periosteum of patients in the study group compared to healthy controls. There was also a considerable increase in TOS in the posttraumatic mandibular periosteum of study group patients compared to the controls. The decreased OSI value in patients with mandibular fracture compared to the control group was also observed. The FRAP level in the mandibular periosteum of the study group patients was significantly higher than in the control group. No statistically relevant changes in plasma TAC, TOS, OSI, and FRAP between the groups were demonstrated (Tables 2 and 3).

### 3.3. Oxidative Damage Products

There was a statistically significant increase in AGE fluorescence in the mandibular periosteum of the study group compared to AGE fluorescence in the control group. AOPP concentration in the mandibular periosteum of the study group was considerably higher than in the control group. There was a statistically relevant increase in the level of 4-HNE in the mandibular periosteum of patients with mandibular fracture compared to healthy controls. The content of AGE, AOPP, 4-HNE, and 8-OHdG in plasma of the study and control group was similar. The concentration of 8-OHdG in the mandibular periosteum of the traumatic patients yielded similar values to those obtained in mandibular periosteum in the control group (Tables 2 and 3).

### 3.4. Correlations

The results of all statistically significant correlations are presented in Table 4. Importantly, we have demonstrated a positive correlation between TAC concentration in the mandibular periosteum and UA level in plasma of patients in the study group. In this group, TOS concentration in the mandibular periosteum was positively correlated with CAT activity in erythrocytes as well as 8-OHdG level in the mandibular periosteum with GPx activity in erythrocytes.

## 4. Discussion

Our findings point to an increased occurrence of redox homeostasis disorders and oxidative stress in patients with mandible fractures treated surgically with titanium miniplates and screws. Although exposure to Ti6Al4V titanium alloy does not alter the clinical picture, the periosteum that contacted the titanium fixations of the mandible showed significantly higher concentrations of oxidation protein products and more extensive lipid modification compared to the control group. We also observed significant changes in the activity/concentration of enzymatic and nonenzymatic antioxidants in the periosteum as well as in the plasma and erythrocytes of the study group.

Titanium is widely used in many fields of medicine and dentistry due to its good biotolerance (biocompatibility) and its osseointegration ability to foster the contact thickening of bones [22]. Titanium and its alloys are used in the production of joint implants and external stabilizers as well as other metal components such as plates, clamps, screws, clips, dental implants, artificial valves, and vascular stents [22]. Their unique advantage is that they do not require removal for at least 20 years. Titanium miniplates and screws are also basic biomaterials used in the surgical treatment of mandibular fractures. One of the most commonly used titanium fixations in maxillofacial surgery is the Ti6Al4V alloy containing, in addition to titanium, 6% aluminium (Al) and 4% vanadium (V) [23]. Ti6Al4V demonstrates high mechanical strength and corrosion resistance due to the presence of a tight protective passive layer of titanium dioxide (TiO_2_) on the surface of the alloy [9, 24]. It is believed that the passive layer effectively protects the surface of the implant from the effects of corrosive agents present in the environment of body fluids [25]. However, there are reports stating that in the period from the implantation to the exploitation of the biomaterial, the TiO_2_ layer may be damaged, resulting in the release of metal ions (such as titanium, aluminium, or vanadium) into the surrounding tissues and organs [9]. In physiological conditions, the body content of these trace elements is very low. Corrosion of the implant, however, raises their concentration and may thus interfere with the process of their natural degradation. The local interaction of metal ions or products of the corrosion of metallic materials with the body tissues is referred to as metallosis [26].

The mandible is the only moving bone of the facial part of the skull. In patients with titanium implants, mandibular movements triggered by strong muscles cause micromovements of the fixed bone fragments, which increases the friction between the miniplates and the screws. This phenomenon is responsible for the corrosion of titanium fixations and the release of titanium ions to the tissues/organs surrounding the implants [3]. In our investigation, the deposition of titanium particles in the study group was observed in the form of gray periosteal discolourations adjacent to the titanium miniplates and screws (data not shown). Importantly, none of the patients had any symptoms of chronic and acute inflammation or healing disorders at the site of the fracture. All patients had their metal implants removed exclusively for reasons not connected to a health risk. Indeed, the most common reason for the removal of miniplates and screws (14 patients) was the desire to get rid of nonfunctional implants in order to avoid future reactions to a foreign body and to eliminate the risk of artefacts causing problems in the interpretation of CT and MRI.

Titanium belongs to the group of biocompatible elements that usually do not cause negative bodily reactions. However, in some people, titanium triggers allergic symptoms (local eczema, rush, and pruritus) [27] and is responsible for the periapical reaction (in the implant-tissue interlayer), which may lead to the destabilization of implants [28]. However, the mechanisms of titanium toxicity in the body are still not well known. Titanium is believed to be responsible for an increased production of oxygen-free radicals and RNS [9, 29]. This element may thus contribute to the induction of oxidative stress as well as structural and functional damage of the cells by the oxidation reaction. Oxidative stress results from overproduction of ROS and/or inadequate activity of the body's antioxidant systems [8, 30]. The products of the oxidative modification of proteins, lipids, and nucleic acids that emerge due to oxidative stress are directly responsible for the damage to the tissues and organs of the body [8]. A significant role in the prevention of oxidative stress is attributed to enzymatic (CAT, SOD, and GPx) and nonenzymatic antioxidants (e.g., UA, reduced glutathione, and vitamin C). These compounds inhibit the formation of free oxygen radicals and/or participate in their conversion into nonreactive derivatives [31]. The antioxidant enzymes are characterized by a greater selectivity of action, while the remaining antioxidants scavenge free radicals in a nonselective manner and/or interrupt the oxidation reaction in the cell [32]. Additionally, nonenzymatic antioxidants may affect the activity of antioxidant enzymes so that the action of one group of antioxidants depends on the other and vice versa [33]. SOD is responsible for the dismutation of the superoxide anion to hydrogen peroxide, which is then broken down by CAT and GPx enzymes. An important role in the defence against free radical overproduction is also carried out by UA, the main blood antioxidant, which constitutes 70–80% of the total antioxidant capacity [34].

The increased activity/concentration of antioxidants (↑SOD, ↑UA, ↑TAC, and ↑FRAP) in the mandibular periosteum of patients with titanium mandibular fixations, compared to the controls, suggests an adaptive reaction of the body in response to excessive ROS production (↑TOS) due to exposure to Ti6Al4V titanium alloy and its wear products. As Wang et al. [25] demonstrated, titanium ions released from implants may stimulate phagocytes (mainly macrophages) and osteoclasts to produce increased amounts of ROS and RNS. By participating in phagocytosis, they can also increase the activity of NADPH oxidase (NOX) [35] which is the main source of free radicals in the cell. High concentrations of ROS can initiate inflammation at the implantation site (by increasing the production of proinflammatory cytokines), which additionally (by positive feedback) increases the production of free oxygen radicals [5, 9, 29]. In our study, the induction of antioxidant defence mechanisms was observed not only in the periosteum but also in the plasma (↑UA) and erythrocytes (↑CAT) of the patients in the study group. These changes point not only to redox imbalances in the implantation site (mandibular periosteum) but also to systemic disorders (blood). A positive correlation found between TAC levels in the mandibular periosteum and the UA concentration in the plasma in the patients from the study group may indicate a relationship between the local and the central antioxidant response. These observations, however, require further research, especially on a larger number of patients with titanium mandibular fixations. The assessment of white cell populations and cytokines produced by oxidative stress may also be helpful for clarifying the systemic etiology of redox imbalance in these patients.

Interestingly, we have observed a significantly lower CAT activity in the periosteum of the patients in the study group versus healthy controls. The decrease in CAT activity may be explained by the exhaustion of the ability to neutralize hydrogen peroxide in the conditions of an overproduction of this tissue. In patients with titanium fixations of the mandible, a higher production of H_2_O_2_ may occur as the direct effect of titanium on the periosteal cells [5, 6, 33] or due to an increase in SOD activity that produces a large amount of hydrogen peroxide as a by-product of the dismutation reaction. This is confirmed by the results of our study (↑SOD in the mandible of the patients in the study group) and may be partially explained by the lack of changes in GPx specific activity.

Despite the increased defence capacity of the antioxidant systems (↑SOD, ↑UA, ↑TAC, and ↑FRAP), we can observe an increased oxidative damage of proteins (↑AGE and ↑AOPP) and lipids (↑4-HNE) in the mandibular periosteum contacting Ti6Al4V titanium alloy. These changes suggest that the body is not able to protect itself effectively against oxidative stress and free radical damage of cells at the site of implantation of miniplates and screws. Importantly, the observed increase in oxidative stress biomarkers did not depend on the time span between osteosynthesis and the removal of the mandibular fixations.

Redox balance disorders as well as products of oxidative damage to the cell components can have a negative impact on the bone remodelling process in patients with mandibular fractures [9, 36]. The proper healing of a fracture depends on the recruitment and differentiation of osteoprogenitor cells towards osteoblasts and osteoclasts. Therefore, this process depends on the production of components of the extracellular matrix of the bone, particularly collagen proteins [37]. It is well known that bone tissue, periosteum, bone marrow, and surrounding soft tissues are involved in the healing of bone fractures; however, a particular role is attributed to the periosteum [38, 39]. Microscopically, the periosteum consists of two layers: the external fibrous layer and the inner layer adhering to the bone (cambium) [40, 41]. In the outer fibrous layer, two sublayers can be distinguished: a superficial layer containing mainly collagen fibers with fibroblasts, which is rich in blood vessels and nerve fibers, and a deeper layer containing a lot of elastic fibers and collagen, a small number of cells and blood vessels. The outer fibrous layer provides elasticity and flexibility [40, 41]. The cambial layer (cambium) is composed of 3-4 cell layers of mainly osteoblasts and preosteoblastic cells and has osteogenic properties. This layer is responsible for the appearance of lamellar bone during growth and creation of woven bone after a fracture [40–42]. In our investigation, all the patients in the study group were treated surgically using an anatomical setting of bone fragments with miniplates and screws. In the case of surgical treatment of fractures, little or no periosteal response is observed [38, 39]. Therefore, it may be assumed that the observed periosteal changes are mainly caused by exposure to titanium implants after fractures.

Oxidative damage to proteins and lipids that occurs due to the overproduction of ROS is responsible for the structural and functional changes of these biomolecules, and this in turn disrupts normal cellular and tissue homeostasis [36, 43]. Titanium ions and cytokines released by macrophages under the influence of the products of titanium implants degradation cause the generation and activation of fibroblasts and osteoclasts, stimulating osteolysis processes [44, 45]. They may also induce genomic instability in human fibroblast cells, which is of particular importance in young people [26], and can inhibit type I collagen synthesis by osteoblasts [46]. In the case of fracture healing, this condition may lead to an inadequate formation of the organic matrix of the bone and thus impair its biomechanical properties. As shown by Sheikhi et al. [47], oxidized lipids lead to osteoblastogenesis and simultaneously stimulate osteoclast activity.

We did not detect oxidative damage to the cell components in the plasma and erythrocytes of the study group patients, which indicates that, excluding the implantation site, the body is capable of preventing free radical damage caused by oxidative stress. A positive correlation between TOS Man and CAT erythrocytes as well as 8-OHdG Man and GPx erythrocytes proves that there is a correlation between the increased oxidative damage in the mandibular periosteum and the central redox disorders in patients with titanium miniplates and screws.

In conclusion, we have demonstrated the occurrence of redox imbalance as well as oxidative damage in the periosteum surrounding the Ti6Al4V titanium alloy. Changes in antioxidant efficiency in the patients treated with titanium implants were also observed in the plasma/erythrocytes, which proves both local and systemic influence of titanium on the human body. The obtained results indicate the need to improve the miniplates and screws used for osteosynthesis by increasing the thickness of the passive TiO_2_ layer or using new, biodegradable, and biocompatible materials (for instance based on magnesium and its alloys). Supplementation with antioxidants would also be helpful in patients treated with titanium fixations; however, further research is needed in this area.

Our work, apart from the undoubted advantages (careful selection of the group of young people (aged 21–29) with similar types of jaw fractures), also had some limitations. We evaluated only the most commonly used biomarkers of oxidative stress; therefore, the assessment of other parameters may lead to different observations and conclusions. It should also be borne in mind that changes in antioxidant defence as well as increased generation of the products of oxidative damage of proteins and lipids may indicate not only the body's response to the introduction of titanium miniplates and screws but also an ongoing callus remodelling in the course of mandibular fracture healing and the related increased protein metabolism.

## Figures and Tables

**Table 1:** Clinical characteristics of patients with titanium mandibular fixations and healthy controls.

Serum/plasma	Control group					Study group				
	Mean	SD	Geometric mean	Lower 95% CI of mean	Upper 95% CI of mean	Mean	SD	Geometric mean	Lower 95% CI of mean	Upper 95% CI of mean
WBC ×10^3^/*μ*L	7.530	1.958	7.278	6.841	8.219	6.917	1.709	6.756	6.315	13.232
RBC ×10^6^/*μ*L	5.207	0.411	5.193	5.062	5.352	5.070	0.264	5.064	4.977	10.047
HGB g/dL	15.270	1.027	15.240	14.908	15.632	15.320	0.720	15.300	15.067	30.387
HCT %	45.230	2.630	45.160	44.304	46.156	45.550	2.123	45.510	44.803	90.353
MCV fL	87.140	3.817	87.070	85.796	88.484	89.630	3.549	89.570	88.381	178.011
MCHC g/dL	33.760	0.918	33.750	33.437	34.083	33.650	1.524	33.620	33.114	66.764
PLT ×10^3^/*μ*L	230.700	43.800	227.100	215.282	246.118	228.300	23.380	227.300	220.070	448.370
PT s	12.230	0.624	12.220	12.010	12.450	11.660	0.699	11.640	11.414	23.074
INR	0.935	0.077	0.933	0.908	0.962	0.908	0.072	0.906	0.883	1.791
APTT s	27.640	1.468	27.610	27.123	28.157	26.780	3.809	26.550	25.439	52.219
Na^+^ mmol/L	140.700	1.890	140.700	140.035	141.365	139.800	1.184	139.800	139.383	279.183
K^+^ mmol/L	4.363	0.317	4.353	4.251	4.475	4.390	0.215	4.386	4.314	8.704
CRP mg/L	1.050	0.370	1.002	0.920	1.180	1.000	0.548	0.880	0.807	1.807

^∗^Statistical significance *p* < 0.05, study group *versus* control.

**Table 2:** Enzymatic and nonenzymatic antioxidants, antioxidant/oxidant status, and oxidative damage in the mandible (Man) of patients with titanium mandibular fixations and healthy controls.

Man	Control group					Study group				
	Mean	SD	Geometric mean	Lower 95% CI of mean	Upper 95% CI of mean	Mean	SD	Geometric mean	Lower 95% CI of mean	Upper 95% CI of mean
CAT nmol H_2_O_2_/min/mg protein	1.435	0.349	0.973	1.312	2.747	0.605^∗^	0.139	0.272	0.556	1.160
SOD mU/mg protein	136.500	20.240	119.400	129.375	265.875	282.000^∗^	44.750	175.200	266.247	548.247
GPx IU/mg protein	0.024	0.003	0.022	0.022	0.046	0.027	0.006	0.025	0.025	0.052
UA *μ*g/mg protein	0.155	0.024	0.125	0.146	0.301	0.285^∗^	0.040	0.234	0.271	0.556
GSH *μ*g/mg protein	1.496	0.299	1.178	1.391	2.887	1.121	0.242	0.810	1.036	2.157
TAC *μ*mol/mg protein	67.420	9.123	57.140	64.209	131.629	252.700^∗^	35.150	204.000	240.327	493.027
TOS *μ*mol H_2_O_2_ Equiv./mg protein	1.088	0.186	0.895	1.023	2.111	1.953	0.632	1.202	1.730	3.683
OSI %	1.956	0.406	1.441	1.813	3.769	1.188	0.337	1.340	1.069	2.257
FRAP *μ*g/mg protein	20.100	3.496	11.540	18.869	38.969	28.660^∗^	4.034	22.820	27.240	55.900
AGE fluorescence/mg protein	91.410	13.390	77.730	86.697	178.107	197.500^∗^	22.010	174.400	189.752	387.252
AOPP *μ*mol/mg protein	1.911	0.242	1.743	1.826	3.737	2.607^∗^	0.246	2.318	2.521	5.128
4-HNE *μ*g/mg protein	234.900	43.450	210.700	219.605	454.505	405.000^∗^	42.540	377.300	390.025	795.025
8-OHdG pg/mg protein	52.340	8.200	46.890	49.454	101.794	71.160	11.740	64.010	67.027	138.187
Total protein mg/mL	3094.000	288.400	2964.000	2992.480	6086.480	1948.000^∗^	210.000	1145.000	1874.078	3822.078

4-HNE: 4-hydroxynonenal protein adducts; 8-OHdG: 8-hydroxydeoxyguanosine; AGE: advanced glycation end products; AOPP: advanced oxidation protein products; CAT: catalase; FRAP: ferric reducing ability of plasma; GPx: glutathione peroxidase; GSH: reduced glutathione; Man: mandible; OSI: oxidative stress index; SOD: superoxide dismutase-1; TAC: total antioxidant status; TOS: total oxidant status; UA: uric acid; ^∗^statistical significance *p* < 0.05, study group *versus* control.

**Table 3:** Enzymatic and nonenzymatic antioxidants, antioxidant/oxidant status, and oxidative damage in the erythrocytes/plasma of patients with titanium mandibular fixations and healthy controls.

Erythrocytes/plasma	Control group					Study group				
	Mean	SD	Geometric mean	Lower 95% CI of mean	Upper 95% CI of mean	Mean	SD	Geometric mean	Lower 95% CI of mean	Upper 95% CI of mean
CAT nmol H_2_O_2_/min/mg protein	6.900	1.047	5.127	6.531	13.431	48.830^∗^	5.823	39.510	46.780	95.610
SOD mU/mg protein	153.600	7.930	149.600	150.809	304.409	154.800	11.270	145.500	150.833	305.633
GPx IU/mg protein	0.020	0.002	0.019	0.019	0.039	0.020	0.001	0.019	0.019	0.039
UA *μ*g/mg protein	0.658	0.011	0.656	0.654	1.312	0.733^∗^	0.012	0.730	0.729	1.462
GSH *μ*g/mg protein	0.687	0.178	0.517	0.625	1.312	0.463	0.044	0.410	0.447	0.910
TAC *μ*mol/mg protein	49.570	7.384	44.020	46.971	96.541	37.680	6.483	26.780	35.398	73.078
TOS *μ*mol H_2_O_2_ Equiv./mg protein	0.629	0.186	0.508	0.564	1.193	0.382	0.055	0.316	0.363	0.745
OSI %	1.318	0.282	1.128	1.219	2.537	1.902	0.551	1.181	1.708	3.610
FRAP *μ*g/mg protein	13.580	3.186	11.750	12.458	26.038	10.990	3.239	6.285	9.850	20.840
AGE fluorescence/mg protein	526.000	30.420	507.700	515.292	1041.292	603.500	180.100	365.200	540.103	1143.603
AOPP umol/mg protein	0.497	0.049	0.452	0.479	0.976	0.506	0.054	0.439	0.487	0.994
4-HNE *μ*g/mg protein	80.890	12.860	70.980	76.363	157.253	108.500	17.570	93.980	102.315	210.815
8-OHdG pg/mg protein	27.350	4.247	21.110	25.855	53.205	32.750^∗^	5.534	27.510	30.802	63.552
Total protein mg/mL	4444.000	109.400	4416.000	4405.490	8849.490	3445.000^∗^	213.300	2712.000	3369.916	6814.916

4-HNE: 4-hydroxynonenal protein adducts; 8-OHdG: 8-hydroxydeoxyguanosine; AGE: advanced glycation end products; AOPP: advanced oxidation protein products; CAT: catalase; FRAP: ferric reducing ability of plasma; GPx: glutathione peroxidase; GSH: reduced glutathione; OSI: oxidative stress index; SOD: superoxide dismutase-1; TAC: total antioxidant status; TOS: total oxidant status; UA: uric acid; ^∗^statistical significance *p* < 0.05, study group *versus* control.

**Table 4:** Correlations of redox biomarkers in control group and patients with titanium mandibular fixations.

		*r*	*p*
*Control group*			
TOS Man	OSI Man	0.551	0.033
TAC Man	UA plasma	0.425	0.043
GPx erythrocytes	FRAP plasma	0.737	0.037
TAC plasma	GSH plasma	0.608	0.003
8-OHdG plasma	AOPP plasma	0.559	0.047
*Study group*			
UA Man	FRAP Man	0.519	0.019
TAC Man	AGE Man	0.450	0.031
4-HNE Man	OSI Man	0.592	0.043
8-OHdG Man	AGE Man	0.883	0.000
TOS Man	OSI Man	0.567	0.034
AGE Man	GPx erythrocytes	0.574	0.025
TOS Man	CAT erythrocytes	0.515	0.043
8-OHdG Man	GPx erythrocytes	0.781	0.002
TAC Man	UA plasma	0.425	0.043
4-HNE Man	GSH plasma	0.662	0.019
GSH Man	GSH plasma	0.417	0.048
SOD erythrocytes	AGE plasma	0.470	0.020
TAC plasma	AOPP plasma	0.695	0.000
TOS plasma	AOPP plasma	0.529	0.000
TOS plasma	GSH plasma	0.645	0.002
8-OHdG plasma	GSH plasma	0.589	0.044

4-HNE: 4-hydroxynonenal protein adducts; 8-OHdG: 8-hydroxydeoxyguanosine; AGE: advanced glycation end products; AOPP: advanced oxidation protein products; CAT: catalase; FRAP: ferric reducing ability of plasma; GPx: glutathione peroxidase; GSH: reduced glutathione; Man: mandible; OSI: oxidative stress index; SOD: superoxide dismutase-1; TAC: total antioxidant status; TOS: total oxidant status; UA: uric acid.
